# Use of Deep Learning to Predict Acute Kidney Injury After Intravenous Contrast Media Administration: Prediction Model Development Study

**DOI:** 10.2196/27177

**Published:** 2021-10-01

**Authors:** Donghwan Yun, Semin Cho, Yong Chul Kim, Dong Ki Kim, Kook-Hwan Oh, Kwon Wook Joo, Yon Su Kim, Seung Seok Han

**Affiliations:** 1 Department of Biomedical Sciences Seoul National University College of Medicine Seoul Republic of Korea; 2 Department of Internal Medicine Seoul National University College of Medicine Seoul Republic of Korea

**Keywords:** acute kidney injury, artificial intelligence, contrast media, deep learning, machine learning, kidney injury, computed tomography

## Abstract

**Background:**

Precise prediction of contrast media–induced acute kidney injury (CIAKI) is an important issue because of its relationship with poor outcomes.

**Objective:**

Herein, we examined whether a deep learning algorithm could predict the risk of intravenous CIAKI better than other machine learning and logistic regression models in patients undergoing computed tomography (CT).

**Methods:**

A total of 14,185 patients who were administered intravenous contrast media for CT at the preventive and monitoring facility in Seoul National University Hospital were reviewed. CIAKI was defined as an increase in serum creatinine of ≥0.3 mg/dL within 2 days or ≥50% within 7 days. Using both time-varying and time-invariant features, machine learning models, such as the recurrent neural network (RNN), light gradient boosting machine (LGM), extreme gradient boosting machine (XGB), random forest (RF), decision tree (DT), support vector machine (SVM), κ-nearest neighbors, and logistic regression, were developed using a training set, and their performance was compared using the area under the receiver operating characteristic curve (AUROC) in a test set.

**Results:**

CIAKI developed in 261 cases (1.8%). The RNN model had the highest AUROC of 0.755 (0.708-0.802) for predicting CIAKI, which was superior to that obtained from other machine learning models. Although CIAKI was defined as an increase in serum creatinine of ≥0.5 mg/dL or ≥25% within 3 days, the highest performance was achieved in the RNN model with an AUROC of 0.716 (95% confidence interval [CI] 0.664-0.768). In feature ranking analysis, the albumin level was the most highly contributing factor to RNN performance, followed by time-varying kidney function.

**Conclusions:**

Application of a deep learning algorithm improves the predictability of intravenous CIAKI after CT, representing a basis for future clinical alarming and preventive systems.

## Introduction

Computed tomography (CT) using contrast media is necessary to clinically detect abnormalities, but the administration of contrast media can lead to acute kidney injury (known as contrast media–induced acute kidney injury [CIAKI]). This is a critical issue due to subsequent risk of irreversible kidney dysfunction and increased mortality [1]. This adverse relationship is more critical in intra-arterial administration of contrast media than in intravenous administration [2]. Nevertheless, frequent use of CT scanning with intravenous contrast media increases the risk of nephrotoxicity, which requires prophylaxis and monitoring of kidney functions [3]. Prediction of intravenous CIAKI after CT scanning may be clinically essential to prepare for intervention in advance, but most relevant studies have primarily focused on intra-arterial CIAKI [4]. Models generated in some studies have predicted intravenous CIAKI, but these models had limitations because model performance was evaluated using a training set (rather than a test set) [5-10], an updated definition of CIAKI was not used [5-12], a prophylaxis protocol was not described [5,10,11], cases with intra-arterial administration of contrast media were combined in the analysis of intravenous cases [6,9,10], and confounding factors were not sufficiently considered [6-10].

Deep learning algorithms have achieved successful prediction of patient outcomes [13,14], which will change the paradigm of clinical decision making from diagnosis to treatment. Among deep learning algorithms, the recurrent neural network (RNN) can learn and characterize a temporal data set. In the nephrology field, using a time-varying data set of kidney function and vital signs, the predictability of outcomes has improved, such as acute kidney injury [15] and intradialytic complications, which are better than other machine learning (eg, gradient boosting machine) [16] and discrete-time logistic regression [17] models. Precise prediction of intravenous CIAKI may be difficult because multiple conditions have interactive and complex effects on its risk, and heterogeneous features of patients along with fluctuating dynamics of kidney functions before CT scanning may also complicate precise prediction. Herein, we addressed whether an RNN model with a time-varying data set including kidney functions could predict the risk of intravenous CIAKI better than other machine learning or conventional scoring models.

## Methods

### Data Source and Study Patients

A total of 19,628 patients underwent CT scanning with intravenous administration of contrast media at the 1-day-care facility of the Seoul National University Hospital between February 2007 and January 2019. This facility was built for the purpose of monitoring and preventing CIAKI in patients at risk, such as those with reduced kidney function or comorbidities. During admission, patients received hydration with 500 mL of 0.9% saline before and after intravenous administration of contrast media and 1200 mg of *N*-acetylcysteine for 3 days [18,19]. Kidney function was subsequently monitored for 2-7 days after CT scanning. Patients aged less than 18 years (n=5), with end-stage kidney disease (n=335), and no information about serum creatinine levels 28 days before and 7 days after CT scanning (n=5103) were excluded. Accordingly, 14,185 cases were included in the analysis (Multimedia Appendix 1). The institutional review board of the National University Hospital approved the study design (no. H-1812-134-997), which was conducted in accordance with the principles of the Declaration of Helsinki.

### Study Features and Outcomes

Baseline characteristics, such as age, sex, weight, height, comorbidities (eg, coronary artery disease, any cancer, liver cirrhosis, glomerulonephritis, kidney transplantation), protocol of CT scanning and volume of contrast media, vital signs (eg, systolic blood pressure, diastolic blood pressure, heart rate, respiratory rate, and body temperature), and medications (eg, β-blocker, calcium channel blocker, angiotensin-converting enzyme inhibitor, angiotensin receptor blocker, hydrochlorothiazide, spironolactone, furosemide, statin, metformin, sodium-glucose cotransporter 2 inhibitor, dipeptidyl peptidase-4 inhibitor, other oral hypoglycemic agents, and insulin), were collected using the patients’ electronic medical records. Vital signs were measured at the time of admission to the facility. Laboratory findings were measured up to 1 month before CT scanning, and variables such as white blood cell count, hemoglobin, hematocrit, platelet count, cholesterol, albumin, total bilirubin, alkaline phosphatase, aspartate transaminase, alanine transaminase, uric acid, blood urea nitrogen, glucose, calcium, phosphate, sodium, potassium, chloride, and bicarbonate were evaluated. The estimated glomerular filtration rate (eGFR) was calculated using the Chronic Kidney Disease Epidemiology Collaboration equation [20]. Time-varying features included serum creatinine, eGFR, and elapsed times before CT scanning, and time-invariant features included all the other features. The baseline characteristics are summarized in Table 1.

**Table 1:** Baseline characteristics.

Features			Total (n=14,185)		CIAKI^a^ (n=261)		Non-CIAKI (n=13,924)		*P* value^b^
Age (years), mean (range)		67.5 (56.7-78.4)		65.2 (54.1-76.3)		67.6 (56.7-78.5)		<.001	
Male, n (%)		10,952 (77.2)		195 (74.7)		10,757 (77.3)		.33	
Body mass index (kg/m²), mean (range)		24.0 (20.7-27.3)		24.0 (20.4-27.6)		24.0 (20.7-27.3)		.94	
**Type of CT^c^, n (%)**									
	Abdomen and pelvis	4360 (30.7)		73 (28.0)		4287 (30.8)		N/A^d^	
	Liver	3323 (23.4)		90 (34.5)		3233 (23.2)		N/A	
	Urogenital	1330 (9.4)		17 (6.5)		1313 (9.4)		N/A	
	Chest	1004 (7.1)		15 (5.7)		989 (7.1)		N/A	
	Others	4168 (29.4)		66 (25.3)		4102 (29.5)		N/A	
	Contrast media volume (mL), mean (range)	98.3 (82.1-114.6)		99.8 (81.5-118.1)		98.3 (82.1-114.6)		.01	
**Vital signs**									
	Systolic blood pressure (mmHg), median (IQR)	126 (116-138)		130 (117.5-141)		126 (116-138)		.002	
	Diastolic blood pressure (mmHg), median (IQR)	75 (68-83)		78 (70-83.5)		75 (68-83)		.01	
	Heart rate (/min), median (IQR)	68 (61-79)		73 (62-82)		68 (61-79)		<.001	
	Respiratory rate (/min), mean (range)	18.3 (17.5-19.2)		18.3 (17.4-19.1)		18.3 (17.5-19.2)		.33	
	Body temperature (°C), mean (range)	36.4 (36.1-36.7)		36.4 (36.1-36.8)		36.4 (36.1-36.7)		.12	
**Comorbidities, n (%)**									
	Diabetes mellitus	4870 (34.3)		126 (48.3)		4744 (34.1)		<.001	
	Hypertension	6896 (48.6)		136 (52.1)		6760 (48.5)		.26	
	Coronary arterial disease	1940 (13.7)		28 (10.7)		1912 (13.7)		.16	
	Cancer, any type	11514 (81.2)		220 (84.3)		11294 (81.1)		.19	
	Liver cirrhosis	2253 (15.9)		58 (22.2)		2195 (15.8)		.005	
	Glomerulonephritis	439 (3.1)		13 (5.0)		426 (3.1)		.08	
	Kidney transplantation recipient	224 (1.6)		2 (0.8)		222 (1.6)		.29	
**Medication, n (%)**									
	Antihypertensive agents	5464 (38.5)		112 (42.9)		5352 (38.4)		.14	
	Diuretics	1905 (13.4)		71 (27.2)		1834 (13.2)		<.001	
	Statins	2731 (19.3)		59 (22.6)		2672 (19.2)		.17	
	Hypoglycemic agents	2553 (18.0)		58 (22.2)		2495 (17.9)		.07	
**Blood findings**									
	Hemoglobin (g/dL), median (IQR)	12.2 (10.6-13.7)		11.15 (10.1-12.4)		12.2 (10.7-13.7)		<.001	
	Hematocrit (%), median (IQR)	36.8 (32.4-40.9)		33.6 (30.4-37.95)		36.8 (32.5-41.1)		<.001	
	Albumin (g/dL), median (IQR)	4.1 (3.8-4.3)		3.8 (3.5-4.2)		4.1 (3.8-4.3)		<.001	
	Blood urea nitrogen (mg/dL), median (IQR)	22 (17-27)		25 (19-35)		22 (17-27)		<.001	
	Creatinine (mg/dL), median (IQR)	1.44 (1.25-1.67)		1.58 (1.27-2.01)		1.44 (1.24-1.67)		<.001	
	eGFR^e^ (mL/min/1.73 m²), median (IQR)	47.1 (38.9-56.1)		42.7 (30.4-54.3)		47.2 (38.9-56.1)		<.001	

^a^CIAKI: contrast media–induced acute kidney injury.

^b^*P* values were derived from the chi-square tests for categorical variables and the Student *t*-test or the Mann-Whitney *U* test for continuous variables.

^c^CT: computed tomography.

^d^N/A: not applicable.

^e^eGFR: estimated glomerular filtration rate.

CIAKI was defined as an increase in serum creatinine of ≥0.3 mg/dL within 2 days or ≥50% within 7 days according to the Kidney Disease Improving Global Outcomes guideline [21]. In a sensitivity analysis, the other definition recommended by the European Society of Urogenital Radiology was used, such as an increase in serum creatinine of ≥0.5 mg/dL or ≥25% within 3 days [22]. As a long-term outcome, information about kidney progression (ie, doubling of serum creatinine, >50% decrease in eGFR, and the need for dialysis and transplantation) and all-cause mortality were obtained using the patients’ electronic medical records, the Korean end-stage renal disease registry, and the National Database of Statistics, Korea.

### Model Development

**Figure 1:**
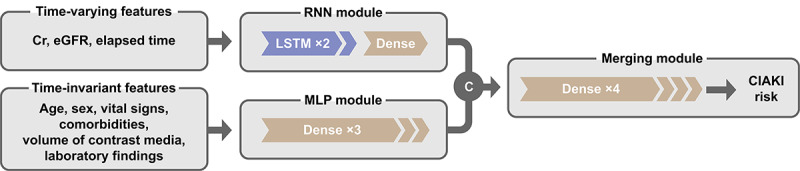
Schematic diagram of the recurrent neural network. C: concatenate; CIAKI: contrast media–induced acute kidney injury; Cr: creatinine; Dense: dense layer; LSTM: long short-term memory; MLP: multilayer perceptron; eGFR: estimated glomerular filtration rate; RNN: recurrent neural network.

Patients were randomly assigned into a training set (70%) to develop the model and a test set (30%) to examine the performance of the model, wherein the occurrence of CIAKI was evenly distributed between the two sets. To develop the RNN model, we combined RNN and multiplayer perceptron (MLP) components. As an RNN component, we used the long short-term memory (LSTM) architecture, which is composed of input, output, and forget gates [23]. The median number of time-varying serum creatinine/eGFR values was 16 during the median timeframe of 4 years (1-9 years) before CT scanning. With respect to these results, 16 consecutive time-varying features were used in the RNN model. These features entered stacked cells and a subsequent dense layer (ie, RNN module), while time-invariant features were processed by 3 dense layers of the MLP module. The results were finally concatenated and then passed through 4 dense layers as a merging module. A dropout layer (rate=0.5) was followed behind each dense layer, while internal LSTM layers used input dropout (rate=0.5) and recurrent dropout (rate=0.5) [24]. Batch normalization layers were located at the end of RNN and multilayer perceptron modules and after the first and third layers of the merging module. Binary cross-entropy loss was used as a loss function to calculate the difference between actual and predicted labels. The Adam method was used for an optimizer [25], and the best parameter was selected using 10-fold cross-validation. Figure 1 presents the schematic diagram of the RNN model. To provide the model training process, we have added the Python code in Multimedia Appendix 2. The script includes data preprocessing, splitting, modeling, and training process information.

We also developed other machine learning models, such as a light gradient boosting machine (LGM), an extreme gradient boosting machine (XGB), a random forest (RF), a decision tree (DT), a support vector machine (SVM), a κ-nearest neighbor, and logistic regression, to compare their performance to the RNN model. These models could not handle time-varying features; therefore, only time-invariant features were included in the models. Tenfold cross-validation was used in the hyperparameter-tuning process, and candidate hyperparameters are listed in Multimedia Appendix 3.

### Feature Importance

Feature importance in the performance of the RNN model was evaluated using SHapley Additive exPlanations (SHAP) [26]. This method explains the model outcome as a sum of values attributed to each input feature, allowing the SHAP value to be interpreted as feature importance. The gradient SHAP model was applied to calculate the SHAP value [26]. The sum of SHAP values was used in the case of time-varying features. For non-RNN models, LinearExplainer (logistic regression and SVM) and TreeExplainer (DT, RF, XGB, and LGM) were used [26].

### Statistical Analysis

Categorical and continuous variables are expressed as proportions and the means ± SD if they had a normal distribution and as medians with IQRs if they were non-normally distributed. Missing values of time-invariant features (4219 cases [28.5%] had at least 1 missing value) were imputed by the κ-nearest-neighboring imputer based on information in the training set [27]. If there were missing values in time-varying features (7031 cases [49.6%] had at least 1 missing value), masking was used during training of the RNN model. Model performance was evaluated in the test set using the area under the receiver operating characteristic curve (AUROC) and compared between models using the Delong test. All *P* values were set as two-sided, and values less than 0.05 were defined as significant. Statistical analyses were performed using R software (version 4.0.2; The Comprehensive R Archive Network: http://cran.r-project.org) and Python (version 3.8.3; Python Software Foundation: http://www.python.org). TensorFlow 2.3.0 (Google Brain, Google Inc.) was used as a deep learning framework [28], and other machine learning algorithms were performed by Scikit-learn [29].

## Results

### Baseline Characteristics

The mean age of cases was 67.5 (SD 11.1) years, and 22.8% (n=3233) were female. The median values of serum creatinine and eGFR were 1.4 mg/dL (IQR 1.3-1.7 mg/dL) and 47.1 mL/min/1.73 m² (IQR 38.9-56.1 mL/min/1.73 m²), respectively. The most common protocol was CT of the abdomen and pelvis (n=4360, 30.7%), followed by the liver (n=3323, 23.4%) and urogenital area (n=1330, 9.4%). Other baseline characteristics of the patients are presented in Table 1. The values of baseline characteristics did not differ between the training and test sets (Multimedia Appendix 4).

### CIAKI and Long-Term Outcomes

Intravenous CIAKI occurred in 261 (1.8%) patients after CT scanning (1.8% in the training set and 2.0% in the test set). During the median follow-up period of 4 years (IQR 2-7 years), renal progression and all-cause mortality were identified in 3400 (24.0%) and 3762 (26.5%) patients, respectively. The CIAKI group had a higher risk of these outcomes compared with the non-CIAKI group (*P*<.001 for renal progression and *P*=.042 for all-cause mortality; see Multimedia Appendix 5).

### Model Performance

**Figure 2:**
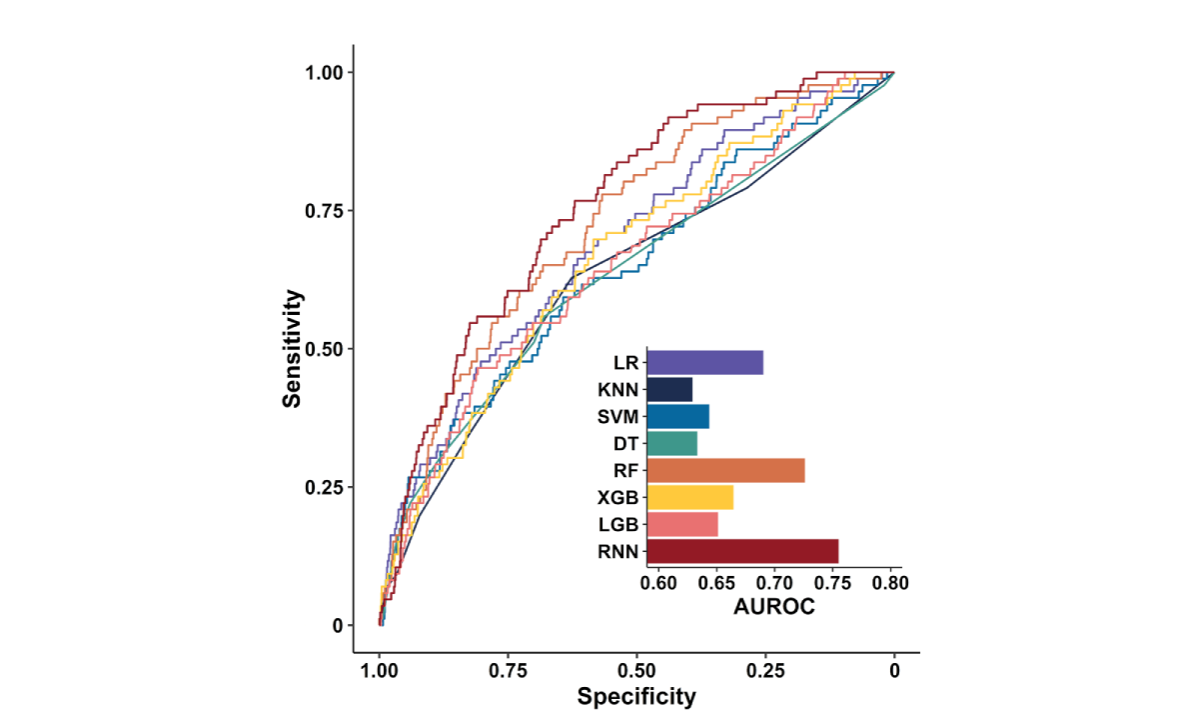
AUROC for predicting intravenous CIAKI in the machine learning models. AUROC: area under the receiver operating characteristic curve; CIAKI: contrast media–induced acute kidney injury; DT: decision tree; KNN: κ-nearest neighbor; LGM: light gradient boosting machine; LR: logistic regression; SVM: support vector machine; RF: random forest; RNN: recurrent neural network; XGB: extreme gradient boosting machine.

When model performance was evaluated in the test set, the RNN model achieved the highest AUROC of 0.755 (95% confidence interval [CI] 0.708-0.802), followed by the RF (0.726 [95% CI 0.674-0.778]) and logistic regression (0.690 [95% CI 0.632-0.748]) (Table 2). The AUROC of the RNN model was greater than that obtained from other machine learning models (*P*<.05), except the RF, and the corresponding curves support these results (Figure 2).

**Table 2:** AUROC^a^ of machine learning models in predicting intravenous CIAKI^b^.

Models	AUROC (95% CI^c^)	*P* value^d^
Logistic regression	0.690 (0.632-0.748)	.01
κ-Nearest neighbor	0.629 (0.566-0.693)	<.001
SVM^e^	0.644 (0.580-0.707)	<.001
DT^f^	0.633 (0.573-0.694)	<.001
RF^g^	0.726 (0.674-0.778)	.17
XGB^h^	0.665 (0.607-0.722)	.006
LGM^i^	0.651 (0.589-0.713)	<.001
RNN^j^	0.755 (0.708-0.802)	N/A^k^

^a^AUROC: area under the receiver operating characteristic curve.

^b^CIAKI: contrast media–induced acute kidney injury.

^c^CI: confidence interval.

^d^Compared to the receiver operating characteristic curve of the RNN model.

^e^SVM: support vector machine.

^f^DT: decision tree.

^g^RF: random forest.

^h^XGB: extreme gradient boosting machine.

^i^LGM: light gradient boosting machine.

^j^RNN: recurrent neural network.

^k^N/A: not available.

We further compared the performance of the RNN model with other published scoring models. Eight studies have developed models to predict intravenous CIAKI [5-12]. The flowchart of study selection and their associated information is presented in Multimedia Appendix 6 and Table 3, respectively. Of these 8 models, 5 used specific features to develop models, such as cystatin C [6-8,10], homocysteine [7], neutrophil gelatinase-associated lipocalin [10], β2-microglobulin [10], and urine output [9]. Accordingly, 3 other models, such as the Mehran score [30], which was originally developed for patients undergoing intra-arterial administration of contrast media during coronary angiography but had also undergone CT scanning in 1 study [11], and two logistic regression–based models without testing of an independent data set [5,12], were compared to the RNN model. The performance of these 3 models was lower than that of the RNN model with the following AUROCs: 0.521 (*P*<.001) in the Mehran score and 0.539 (*P*<.001) and 0.645 (*P*=.022) in the other 2 logistic regression-based models.

**Table 3:** Previous studies predicting intravenous CIAKI^a^.

Studies	Study subjects	CIAKI definition	CIAKI (%)	Prophylaxis protocol	Patients, n (training/test)	Features, n	Modeling methods	AUROC^b^ in test set
Kim et al [5]	Abdominal CT^c^ in emergency department	≥0.5 mg/dL or ≥25% within 3 days	4.5	Not declared	750/0	2	Nomogram	N/A^d^ (0.794 in training set)
Wacker-Gussmann et al [6]^d^	CAG^e^ or CT in hospitalized patients with sCr^f^ levels between 0.8 and 1.3 mg/dL	≥0.5 mg/dL or ≥25% within 48 h	14.2	Oral fluid intake, 2 L	373/0	2	Baseline ratio of CysC^g^/Cr	N/A (0.826 in training set)
Li et al [7]	Coronary CT in patients with eGFR^h^ of ≥60 mL/min/1.73 m^2^	≥0.5 mg/dL or ≥25% within 48 h	9.8	Oral fluid intake, 500 mL	580/0	5	AUROC with single feature	N/A (0.829 of homocysteine in training set)
Li et al [8]	Coronary CT in patients with eGFR of ≥60 mL/min/1.73 m^2^	≥0.5 mg/dL or ≥25% within 48 h	12.3	Oral fluid intake, 500 mL	424/0	2	AUROC with single feature	N/A (0.781 of CysC in training set)
Hocine et al [9]^i^	CAG or CT in intensive care unit	≥0.5 mg/dL or ≥25% within 3 days	60.1	No routine protocol	149/0	1	RIFLE^j^ criteria	N/A
Ho et al [11]	CT pulmonary angiogram in intensive care unit	>0.5 mg/dL within 48 h	40.9	Not declared	0^a^/137	8	Mehran score	0.864
Jeon et al [12]	CT in cancer patients with eGFR of <45 mL/min/1.73 m^2^	>25% within 2-6 days	2.46	0.9% Saline with *N*-acetylcysteine	2185/539	3	Scoring system based on logistic regression	0.749
Banda et al [10]^k^	CAG and CT in hospitalized patients	>0.5 mg/dL or >25% within 48-72 h	N/A	Not declared	90/0	5	AUROC with single feature	N/A (0.684 of β2-microglobulin in training set)

^a^CIAKI: contrast media–induced acute kidney injury.

^b^AUROC: area under the receiver operating characteristic curve.

^c^CT: computed tomography.

^d^N/A: not available.

^e^CAG: coronary angiography.

^f^sCr: serum creatinine.

^g^CysC: cystatin C.

^h^eGFR: estimated glomerular filtration rate.

^i^Used the Mehran risk score.

^j^RIFLE: Risk Injury Failure Loss of kidney function and End-stage kidney disease classification.

^k^Included patients with both intravenous and intra-arterial administration of contrast media.

### Sensitivity Analysis

For sensitivity analysis, another definition of CIAKI was used, an increase in serum creatinine of ≥0.5 mg/dL or ≥25% within 3 days [22]. The RNN model was the best model in predicting the risk of CIAKI, with an AUROC of 0.716 (95% CI 0.664-0.768), which was greater than that of most of the other machine learning models (Multimedia Appendix 7). The corresponding curves support these results (Multimedia Appendix 8).

Other machine learning models were trained after including 48 features (ie, 16 sets of serum creatinine, eGFR, and elapsed times) as an independent feature without timed order. The results are summarized in Multimedia Appendix 9. Although these features were considered in the models, the model performance was less than that of the RNN model.

Furthermore, the original pipeline was separated into 4 models (MLP alone, MLP plus merging, RNN alone, and RNN plus merging), and their performance was compared with that of the original pipeline (named a default model). The AUROC plots are presented in Multimedia Appendix 10. The deep learning model with the MLP module alone and the RNN module alone had AUROCs of 0.705 (95% CI 0.647-0.763) and 0.702 (95% CI 0.642-0.763), respectively. After adding the merging module to these models, the AUROCs were 0.710 (95% CI 0.653-0.768) in the MLP-plus-merging module and 0.675 (95% CI 0.610-0.740) in the RNN-plus-merging module. All these values were lower than the value from the original deep learning model.

To evaluate the effect of the model complexity on performance, we built other deep learning architectures, such as a simple model (ie, 1 less dense layer in the RNN module, MLP module, and merging module) and a complex model (ie, 1 more dense layer in the RNN module, MLP module, and merging module). The AUROCs were 0.751 (95% CI 0.702-0.801) and 0.734 (95% CI 0.678-0.791) in the simple and complex models, respectively. We also developed models with a single LSTM layer having a simpler RNN architecture (named “single model”) and with two stacked bidirectional LSTM layers having a more complex RNN architecture (named “bidirectional model”). The single and bidirectional models had AUROCs of 0.746 (95% CI 0.696-0.795) and 0.717 (95% CI 0.656-0.777), respectively. The AUROC plots of these models compared to that of the original model (named “default model”) are described in Multimedia Appendix 11.

### Feature-Ranking Analysis

**Figure 3:**
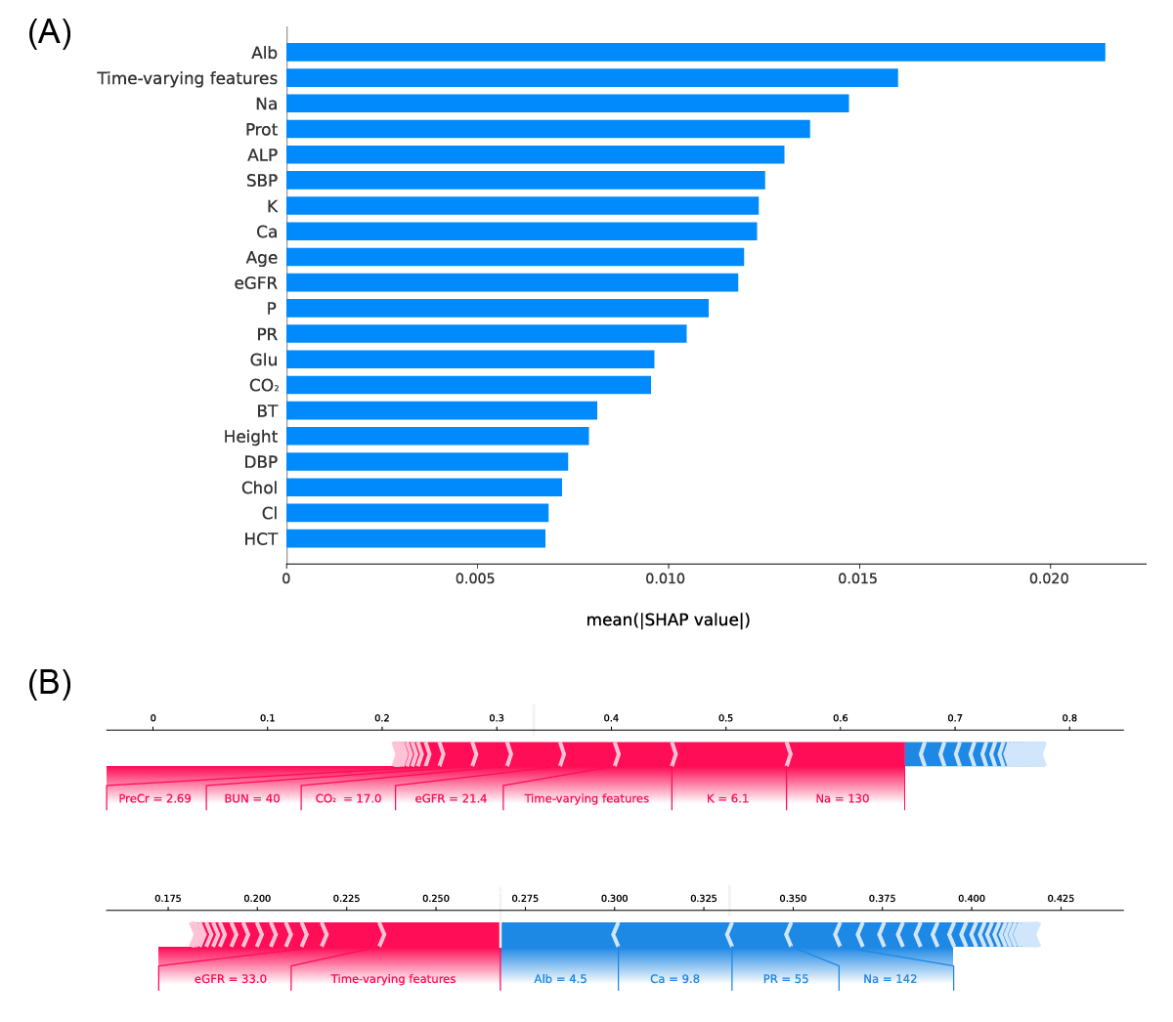
SHAP analysis of the RNN model. (A) Feature ranking according to SHAP value. (B) Two cases to explain the risk of intravenous CIAKI with SHAP values. RNN: recurrent neural network; SHAP: SHapley Additive eXplanations; CIAKI: contrast media–induced acute kidney injury; Alb: albumin; ALP: alkaline phosphatase; BT: body temperature; Ca: calcium; Chol: cholesterol; Cl: chloride; CO_2_: bicarbonate; DBP: diastolic blood pressure; eGFR; estimated glomerular filtration rate; Glu: glucose; HCT: hematocrit: K: potassium; Na: sodium; P: phosphate; PR: pulse rate; PreCr; baseline creatinine; Prot: protein; SBP: systolic blood pressure.

Feature importance in RNN performance was estimated using SHAP (Figure 3A). Serum albumin had the highest impact on model output, and time-varying serum creatinine was ranked second. Age, several laboratory features (eg, sodium, protein, and alkaline phosphatase), and vital signs (eg, systolic blood pressure) were also highly ranked. We also explored SHAP values in non-RNN machine learning models (Multimedia Appendix 12). In the RF model and the LGM model, which achieved the second- and third-highest performance, SHAP values were highly correlated (Pearson’s correlation of the mean of absolute SHAP values=0.781; *P*<0.001; Multimedia Appendix 13), and the time-invariant features with high impact in the RNN model (eg, albumin, sodium, and protein) were also highly ranked.

Figure 3B shows 2 representative cases with CIAKI. The model predicted the risk of CIAKI as 0.680 (true-positive) and 0.264 (false-positive) in the upper and lower cases, respectively. According to SHAP analysis, hyponatremia, hyperkalemia, time-varying serum features, and low eGFR contributed to precise prediction in the upper case. In the lower case, although serum albumin, calcium, and other parameters underestimated the risk of CIAKI, the time-varying features and low eGFR corrected this false prediction.

## Discussion

### Principal Results

Intravenous CIAKI is a critical issue because it contributes to poor outcomes [31], as noted in its association with renal progression and increased mortality above. This study first applied the RNN algorithm to predict intravenous CIAKI with a greater AUROC than that obtained from other machine learning or conventional scoring models. These results indicate that the time-varying data of kidney function (ie, serum creatinine and eGFR) significantly contribute to the precise prediction of intravenous CIAKI. SHAP analysis demonstrated that feature importance could help understand how risk is estimated.

Because kidney function fluctuates over time, a single value of serum creatinine or eGFR may not perfectly represent the kidney function of patients. Certain attempts using time-varying kidney functions by time-dependent Cox regression [32] and trajectory analysis [33] have improved the precise estimation of kidney function. Recently, deep learning with the RNN model showed favorable performance in predicting acute kidney injury [15], implying the additive benefit of time-varying kidney functions to the model performance. Patients with comorbidities, including cancer, diabetes mellitus, and chronic kidney disease, are recommended for frequent follow-up of their kidney function because these data can be used to better predict the trend of kidney function than a single estimation. In this regard, the present RNN model achieved the highest performance in predicting intravenous CIAKI with time-varying features.

Deep learning architecture is complex and difficult to interpret in nature and is referred to as a black box. To overcome this limitation, this study applied SHAP to concretely explain the model output. Using SHAP values, clinicians can comprehend how the risk probability is explained by the results of various features and decide whether the model output is feasible. If the model prediction seems to be imprecise, as in the lower case in Figure 3B, the SHAP values in features highly relevant to the model performance provide room for reconsideration.

### Limitations

Despite these informative results, there are limitations to be discussed. The study design was retrospective and needs to be validated in future independent cohorts. Unidentified factors, such as urine output and heart function, may provide additional information about the risk of CIAKI, but the present data set included most clinically used features. The prophylaxis protocol may differ between centers, and thus, the present RNN model may need to be adjusted when applied externally.

### Conclusions

Application of a deep learning algorithm improves the predictability of intravenous CIAKI, and our model performs better than other machine learning and conventional scoring models. These results may be attributable to the consideration of time-varying kidney functions, in addition to time-invariant features, and corresponding SHAP values may maximize the utility of the model in clinics. If proactive management of intravenous CIAKI is possible via precise prediction, overall patient outcomes will improve. The study results represent the basis of this goal.

## Appendix

Multimedia Appendix 1 Flowchart of data retrieval and splitting.

Multimedia Appendix 2 Python pseudocode including data preprocessing, splitting, modeling, and training processes.

Multimedia Appendix 3 Hyperparameters used in machine learning models.

Multimedia Appendix 4 Baseline characteristics in the training and test sets.

Multimedia Appendix 5 Kaplan-Meier curves of renal survival (A) and patient survival (B) according to intravenous CIAKI. CIAKI: contrast media–induced acute kidney injury.

Multimedia Appendix 6 Flowchart of study selection regarding the modeling of predicting intravenous CIAKI. CIAKI: contrast media–induced acute kidney injury.

Multimedia Appendix 7 Table of AUROCs for predicting intravenous CIAKI, which was defined as an increase in serum creatinine ≥0.5 mg/dL or ≥25% within 3 days. AUROC: area under the receiver operating characteristic curve; CIAKI: contrast media–induced acute kidney injury.

Multimedia Appendix 8 Plots showing AUROCs for predicting intravenous CIAKI, which was defined as an increase in serum creatinine ≥0.5 mg/dL or ≥25% within 3 days. AUROC: area under the receiver operating characteristic curve; CIAKI: contrast media–induced acute kidney injury.

Multimedia Appendix 9 AUROCs of machine learning models in predicting intravenous CIAKI. AUROC: area under the receiver operating characteristic curve; CIAKI: contrast media–induced acute kidney injury.

Multimedia Appendix 10 AUROC for predicting intravenous CIAKI according to the combination of modules. AUROC: area under the receiver operating characteristic curve CIAKI: contrast media–induced acute kidney injury.

Multimedia Appendix 11 AUROC for predicting intravenous CIAKI in RNN models. AUROC: area under the receiver operating characteristic curve; CIAKI: contrast media–induced acute kidney injury; RNN: recurrent neural network.

Multimedia Appendix 12 SHAP analysis of machine learning models. SHAP: SHapley Additive exPlanations.

Multimedia Appendix 13 Scattered plot showing the paired mean of absolute SHAP values of 53 time-invariant features in random forest and LGM models. SHAP: SHapley Additive exPlanations; LGM: light gradient boosting machine.

## Abbreviations

AUROC: area under the receiver operating characteristic curve
CI: confidence interval
CIAKI: contrast media–induced acute kidney injury
CT: computed tomography
DT: decision tree
eGFR: estimated glomerular filtration rate
LGM: light gradient boosting machine
LSTM: long short-term memory
MLP: multiplayer perceptron
RF: random forest
RNN: recurrent neural network
SHAP: SHappley Additive exPlanations
SVM: support vector machine
XGB: extreme gradient boosting machine
