# Correction: Use of Deep Learning to Predict Acute Kidney Injury After Intravenous Contrast Media Administration: Prediction Model Development Study

**DOI:** 10.2196/34411

**Published:** 2021-11-02

**Authors:** Donghwan Yun, Semin Cho, Yong Chul Kim, Dong Ki Kim, Kook-Hwan Oh, Kwon Wook Joo, Yon Su Kim, Seung Seok Han

**Affiliations:** 1 Department of Biomedical Sciences Seoul National University College of Medicine Seoul Republic of Korea; 2 Department of Internal Medicine Seoul National University College of Medicine Seoul Republic of Korea

In “Use of Deep Learning to Predict Acute Kidney Injury After Intravenous Contrast Media Administration: Prediction Model Development Study” (JMIR Med Inform 2021;9(10):e27177), one error was noted.

In the originally published paper, names of 5 authors (Yong Chul Kim, Dong Ki Kim, Kwon Wook Joo, Yon Su Kim, and Seung Seok Han) were inadvertently formatted with middle initials instead of the full author names.

The full authorship list was listed as follows in the originally published paper.

Donghwan Yun, Semin Cho, Yong C Kim, Dong K Kim, Kook-Hwan Oh, Kwon W Joo, Yon S Kim, Seung S Han

This has been corrected to:

Donghwan Yun, Semin Cho, Yong Chul Kim, Dong Ki Kim, Kook-Hwan Oh, Kwon Wook Joo, Yon Su Kim, Seung Seok Han

The correction will appear in the online version of the paper on the JMIR Publications website on November 1, 2021, together with the publication of this correction notice. Because this was made after submission to PubMed, PubMed Central, and other full-text repositories, the corrected article has also been resubmitted to those repositories.

