# Supplementary figures and images for: Use of Deep Learning to Predict Acute Kidney Injury After Intravenous Contrast Media Administration: Prediction Model Development Study
Source: JMIR Med Inform. 2021 Oct 1;9(10):e27177. doi: 10.2196/27177 (PMC8520134; doi:10.2196/27177)

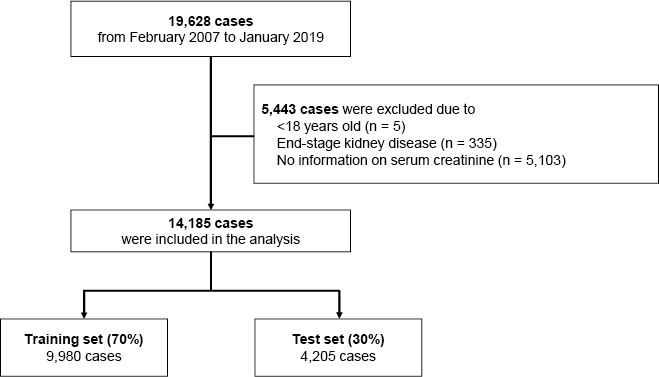

Supplement: Multimedia Appendix 1 [file medinform_v9i10e27177_app1.png]

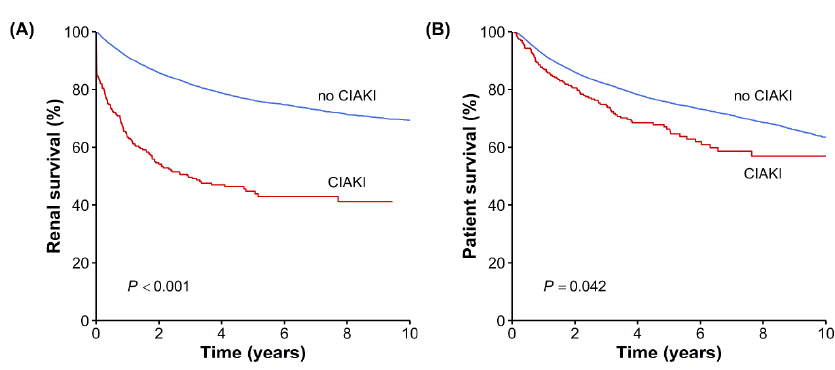

Supplement: Multimedia Appendix 5 [file medinform_v9i10e27177_app5.png]

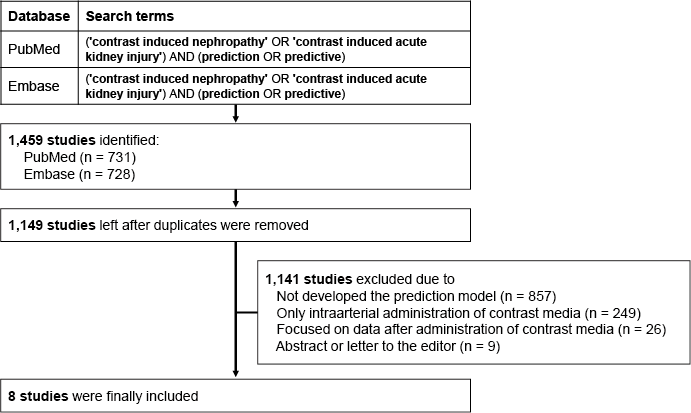

Supplement: Multimedia Appendix 6 [file medinform_v9i10e27177_app6.png]

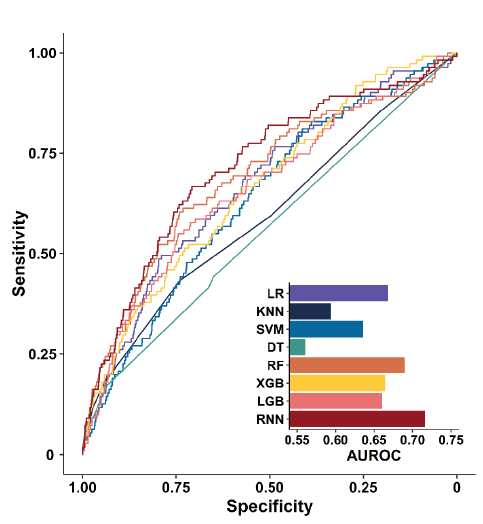

Supplement: Multimedia Appendix 8 [file medinform_v9i10e27177_app8.png]

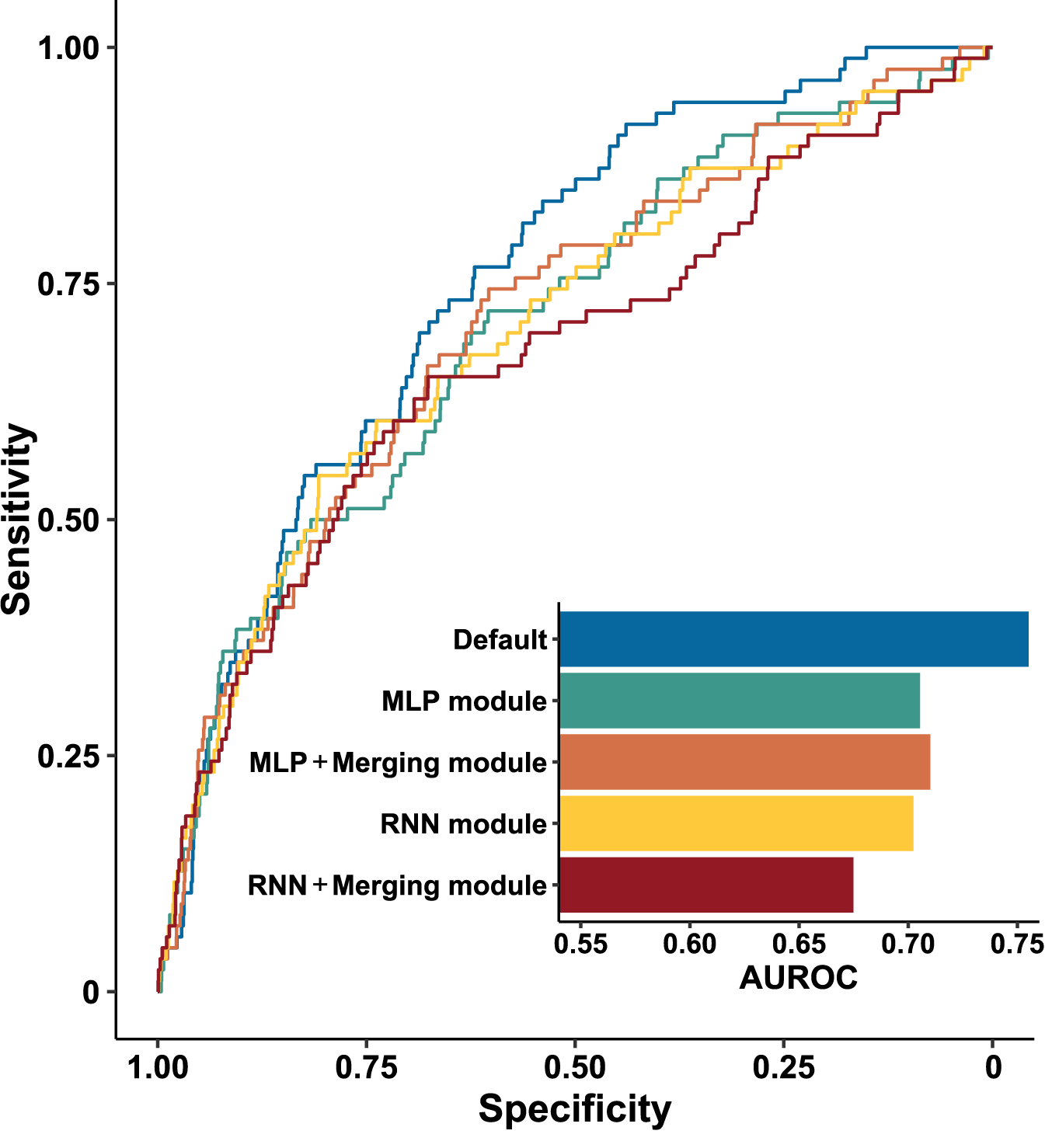

Supplement: Multimedia Appendix 10 [file medinform_v9i10e27177_app10.png]

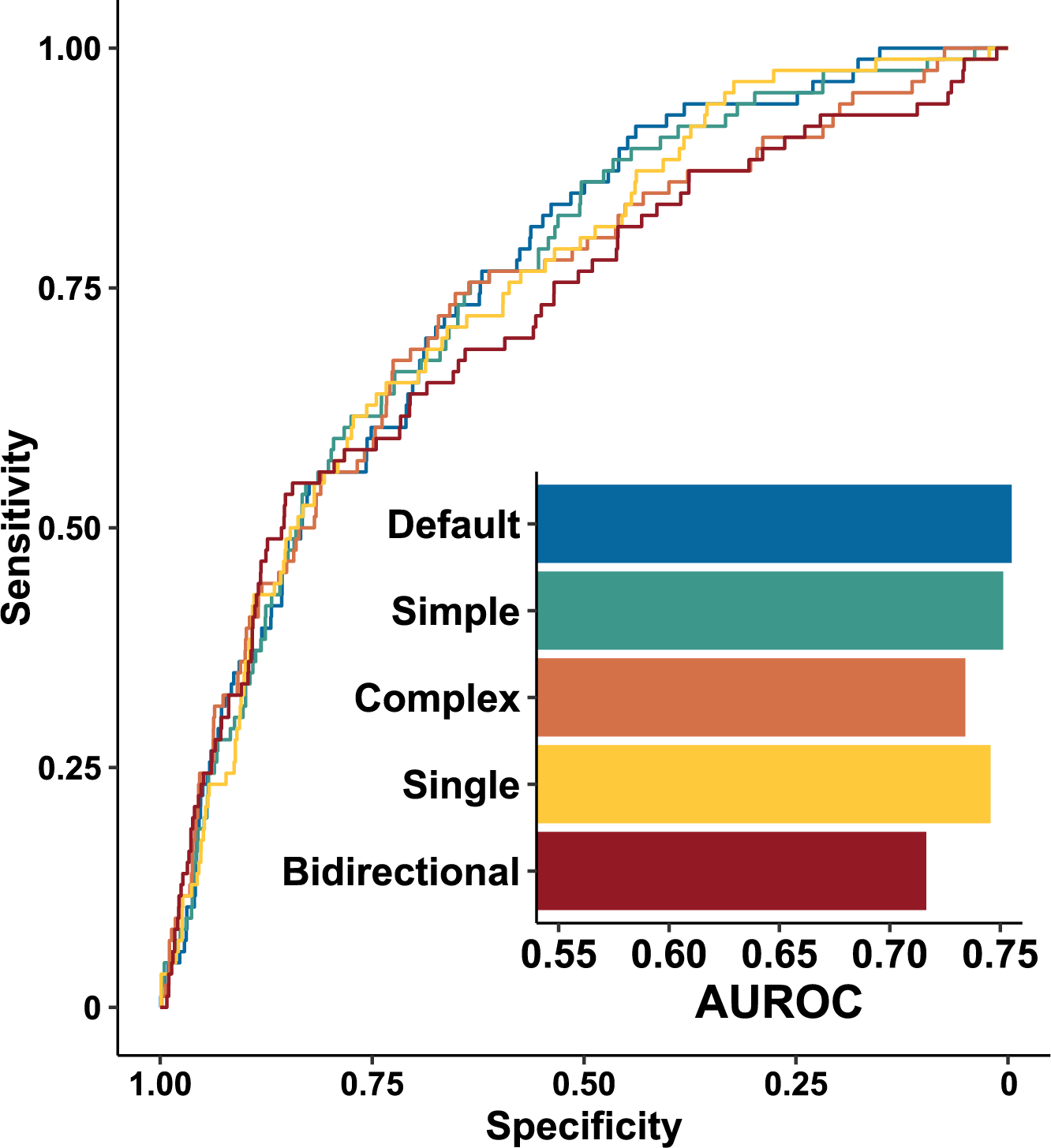

Supplement: Multimedia Appendix 11 [file medinform_v9i10e27177_app11.png]

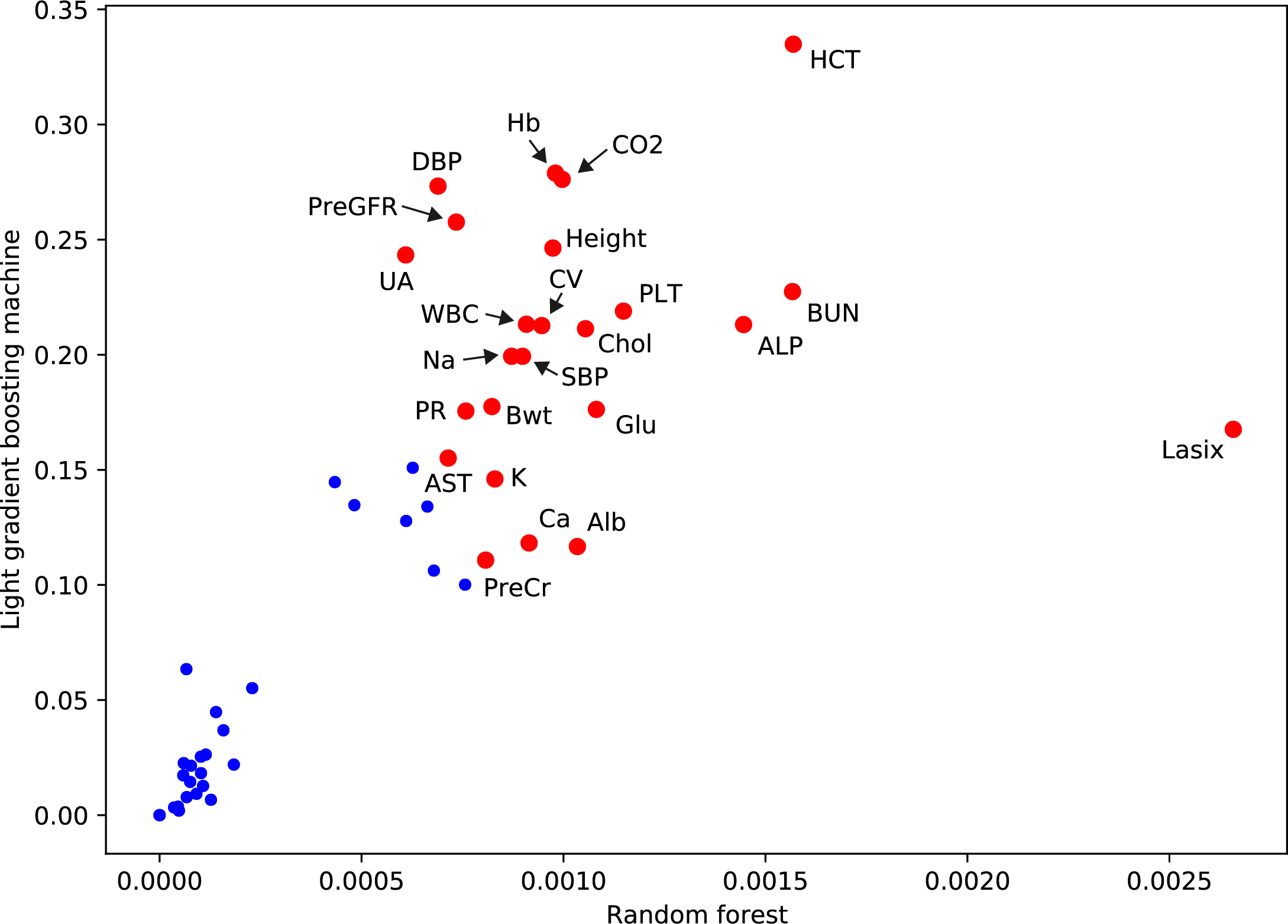

Supplement: Multimedia Appendix 13 [file medinform_v9i10e27177_app13.png]
