# Supplementary material for: Use of Deep Learning to Predict Acute Kidney Injury After Intravenous Contrast Media Administration: Prediction Model Development Study
Source: JMIR Med Inform. 2021 Oct 1;9(10):e27177. doi: 10.2196/27177 (PMC8520134; doi:10.2196/27177)
Supplement: Multimedia Appendix 3 [file medinform_v9i10e27177_app3.docx]

Multimedia Appendix 2. Hyperparameters used in machine learning models

| Models | Hyperparameters |
| --- | --- |
| Logistic regression | penalty (L1, L2)  C (0.01, 0.1, 1, 10) |
| κ-nearest neighbor | n_neighbors (5, 10, 50, 100)  p (1, 2) |
| Support vector machine | kernal (rbf, linear, poly)  C (0.01, 0.1, 1, 10) |
| Decision tree | max_depth (3, 6, 12)  min_samples_split (3, 6, 12) |
| Random forest | criterion (Gini, Entropy)  n_estimators (100, 200, 300)  max_depth (3, 6, 12) |
| Extreme gradient boosting machine | n_estimators (100, 200, 400)  eta (0.1, 0.2)  gamma (0, 0.1, 0.2) |
| Light gradient boosting machine | n_estimators (100, 200, 400)  learning_rate (0.1, 0.2)  num_leaves (31, 62) |
