# Supplementary material for: Use of Deep Learning to Predict Acute Kidney Injury After Intravenous Contrast Media Administration: Prediction Model Development Study
Source: JMIR Med Inform. 2021 Oct 1;9(10):e27177. doi: 10.2196/27177 (PMC8520134; doi:10.2196/27177)
Supplement: Multimedia Appendix 4 [file medinform_v9i10e27177_app4.docx]

Multimedia Appendix 3. Baseline characteristics in the training and test sets

|  | Training set (70%; n = 9,980) | | Test set (30%; n = 4,205) | |
| --- | --- | --- | --- | --- |
| Features | Results | Missing (%) | Results | Missing (%) |
| Age (year) | 67.6 ± 10.9 | 0 | 67.3 ± 10.9 | 0 |
| Male (%) | 78.0 | 0 | 75.4 | 0 |
| Body weight (kg) | 64.8 ± 10.8 | 1.9 | 64.4 ± 10.5 | 1.9 |
| Height (cm) | 164.2 ± 7.8 | 2.3 | 163.8 ± 8.3 | 2.3 |
| Contrast media volume (ml) | 98.6 ± 16.3 | 2.3 | 97.7 ± 16.2 | 2.3 |
| Systolic blood pressure (mmHg) | 127 (116–138) | 2.4 | 126 (116–138) | 2.4 |
| Diastolic blood pressure (mmHg) | 75 (68–83) | 2.4 | 75 (68–83) | 2.4 |
| Heart rate (/min) | 68 (60–79) | 2.4 | 69 (62–80) | 2.4 |
| Respiratory rate (/min) | 18.3 ± 0.8 | 2.6 | 18.3 ± 0.9 | 2.6 |
| Body temperature (°C) | 36.4 ± 0.3 | 11.2 | 36.4 ± 0.3 | 11.2 |
| Baseline creatinine (mg/dl) | 1.43 (1.24–1.66) | 0 | 1.46 (1.26–1.70) | 0 |
| Baseline eGFR (ml/min/1.73 m²) | 47.5 (39.2–56.4) | 0 | 46.3 (37.8–55.2) | 0 |
| White blood cell (×10^3^/ul) | 5.8 (4.6–7.3) | 5.2 | 5.8 (4.5–7.2) | 5.2 |
| Hemoglobin (g/dl) | 12.3 (10.7–13.7) | 3.9 | 12.1 (10.6–13.6) | 3.9 |
| Hematocrit (%) | 37.1 (32.5–41.1) | 4 | 36.4 (32.3–40.7) | 4 |
| Platelet (×10^3^/ul) | 187 (139–235) | 3.1 | 188 (140–239) | 3.1 |
| Cholesterol (mg/dl) | 161 (137–188) | 3.1 | 161 (136–189) | 3.1 |
| Protein (mg/dl) | 7.1 ± 0.6 | 2.8 | 7.1 ± 0.6 | 2.8 |
| Albumin (mg/dl) | 4.0 ± 0.5 | 2.9 | 4.0 ± 0.5 | 2.9 |
| Total bilirubin (mg/dl) | 0.8 ± 0.5 | 2.7 | 0.7 ± 0.5 | 2.7 |
| Alkaline phosphatase (IU/l) | 73 (58–94) | 2.1 | 72 (58–96) | 2.1 |
| Aspartate transaminase (IU/l) | 23 (19–31) | 2 | 23 (18–30) | 2 |
| Alanine transaminase (IU/l) | 19 (13–28) | 2 | 18 (13–27) | 2 |
| Uric acid (mg/dl) | 6.4 (5.3–7.6) | 1.4 | 6.5 (5.4–7.7) | 1.4 |
| Blood urea nitrogen (mg/dl) | 22 (17–27) | 0.2 | 22 (18–28) | 0.2 |
| Glucose (mg/dl) | 106 (94–129) | 2.6 | 105 (93–128) | 2.6 |
| Calcium (mg/dl) | 9.1 (8.7–9.4) | 0.9 | 9.1 (8.7–9.4) | 0.9 |
| Phosphate (mg/dl) | 3.3 (2.9–3.7) | 1.1 | 3.3 (2.9–3.7) | 1.1 |
| Sodium (mmol/l) | 139.6 ± 3.0 | 5.9 | 139.7 ± 3.0 | 5.9 |
| Potassium (mmol/l) | 4.6 ± 0.5 | 7 | 4.6 ± 0.5 | 7 |
| Chloride (mmol/l) | 105.4 ± 3.7 | 6.2 | 105.5 ± 3.8 | 6.2 |
| Bicarbonate (mmol/l) | 24.7 ± 3.5 | 8.9 | 24.6 ± 3.5 | 8.9 |
| Diabetes mellitus (%) | 34.4 | 0 | 34.1 | 0 |
| Hypertension (%) | 48.9 | 0 | 47.9 | 0 |
| Coronary artery disease (%) | 13.9 | 0 | 13.2 | 0 |
| Cancer, any type (%) | 81.6 | 0 | 80.3 | 0 |
| Liver cirrhosis (%) | 15.9 | 0 | 15.8 | 0 |
| Glomerulonephritis (%) | 3.2 | 0 | 2.9 | 0 |
| Kidney transplant recipient (%) | 1.4 | 0 | 2.0 | 0 |
| Beta blocker (%) | 17.1 | 0 | 14.2 | 0 |
| Calcium channel blocker (%) | 20.8 | 0 | 20.7 | 0 |
| ACE inhibitor / angiotensin receptor blocker (%) | 25.2 | 0 | 24.6 | 0 |
| Hydrochlorothiazide (%) | 7.0 | 0 | 6.8 | 0 |
| Spironolactone (%) | 4.1 | 0 | 3.8 | 0 |
| Furosemide (%) | 5.9 | 0 | 5.9 | 0 |
| Statin (%) | 19.2 | 0 | 19.4 | 0 |
| Metformin (%) | 8.7 | 0 | 7.8 | 0 |
| Sodium-glucose co-transporter 2 inhibitor (%) | 0.1 | 0 | 0.2 | 0 |
| Dipeptidyl peptidase-4 inhibitor (%) | 3.6 | 0 | 3.5 | 0 |
| Other oral hypoglycemic agents (%) | 10.9 | 0 | 11.4 | 0 |
| Insulin (%) | 3.8 | 0 | 3.7 | 0 |

Data are expressed as the mean ± standard deviation, median (interquartile range), and percentage for normally distributed continuous, nonnormally distributed continuous, and categorical features, respectively.

ACE, angiotensin converting enzyme.
