# Supplementary material for: Use of Deep Learning to Predict Acute Kidney Injury After Intravenous Contrast Media Administration: Prediction Model Development Study
Source: JMIR Med Inform. 2021 Oct 1;9(10):e27177. doi: 10.2196/27177 (PMC8520134; doi:10.2196/27177)
Supplement: Multimedia Appendix 7 [file medinform_v9i10e27177_app7.docx]

Multimedia Appendix 6. Area under the receiver operating characteristics for predicting intravenous contrast media-induced acute kidney injury*

| Models | AUROC (95% CI) | *P* value^†^ |
| --- | --- | --- |
| Logistic regression | 0.668 (0.616–0.721) | 0.053 |
| κ-nearest neighbor | 0.594 (0.537–0.652) | <0.001 |
| Support vector machine | 0.636 (0.585–0.688) | 0.009 |
| Decision tree | 0.561 (0.510–0.612) | <0.001 |
| Random forest | 0.690 (0.635–0.745) | 0.302 |
| Extreme gradient boosting machine | 0.664 (0.613–0.715) | 0.052 |
| Light gradient boosting machine | 0.661 (0.605–0.716) | 0.028 |
| Recurrent neural network | 0.716 (0.664–0.768) |  |

*Defined as an increase in serum creatinine ≥0.5 mg/dl and/or ≥25% within 3 days.

^†^Compared to the receiver operating characteristic curve of the recurrent neural network model.

AUROC, area under the receiver operating characteristics; CI, confidence interval
