# Supplementary material for: Use of Deep Learning to Predict Acute Kidney Injury After Intravenous Contrast Media Administration: Prediction Model Development Study
Source: JMIR Med Inform. 2021 Oct 1;9(10):e27177. doi: 10.2196/27177 (PMC8520134; doi:10.2196/27177)

Table. Area under the receiver operating characteristics of machine learning models in predicting intravenous contrast media-induced acute kidney injury

| Models | AUROC^a^ (95% CI^b^) | *P* value^c^ |
| --- | --- | --- |
| Logistic regression | 0.697 (0.641-0.753) | .04 |
| κ-nearest neighbor | 0.535 (0.469-0.602) | <.001 |
| Support vector machine | 0.549 (0.490-0.608) | <.001 |
| Decision tree | 0.578 (0.524-0.633) | <.001 |
| Random forest | 0.707 (0.650-0.764) | .07 |
| Extreme gradient boosting machine | 0.679 (0.621-0.737) | .02 |
| Light gradient boosting machine | 0.704 (0.647-0.760) | .10 |
| Recurrent neural network | 0.755 (0.708-0.802) |  |

^a^AUROC: area under the receiver operating characteristics
^b^CI, confidence interval.
^c^Compared to the receiver operating characteristic curve of the RNN model.

Figure. Area under the receiver operating characteristic (AUROC) curve for predicting intravenous contrast media-induced acute kidney injury.


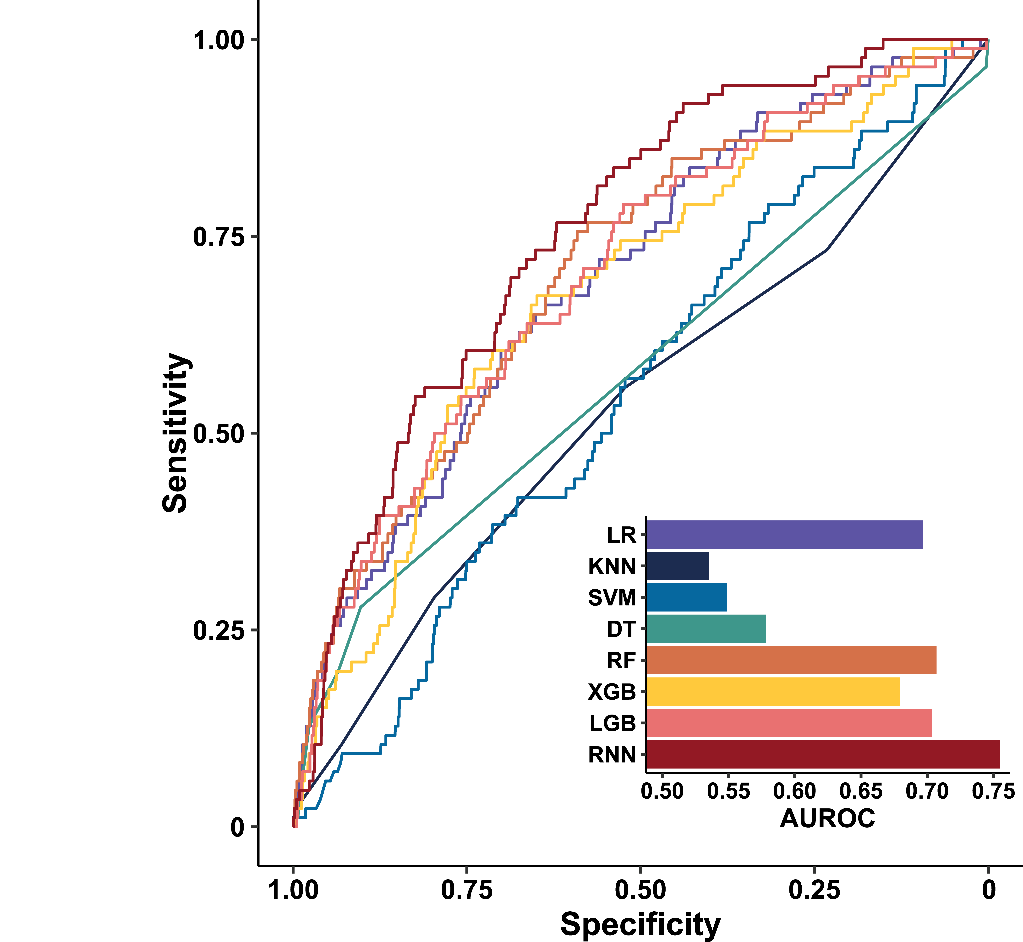

Supplement: Multimedia Appendix 9 [file medinform_v9i10e27177_app9.docx]
