# Supplementary material for: Use of Deep Learning to Predict Acute Kidney Injury After Intravenous Contrast Media Administration: Prediction Model Development Study
Source: JMIR Med Inform. 2021 Oct 1;9(10):e27177. doi: 10.2196/27177 (PMC8520134; doi:10.2196/27177)

Multimedia Appendix 12. SHapley Additive eXplanations (SHAP) analysis of machine learning models. (A), Logistic regression. (B), Support vector machine. (C), Decision tree. (D), Random forest. (E), Extreme gradient boosting machine. (F), Light gradient boosting machine.

Alb, albumin; ALP, alkaline phosphatase; AST, aspartate transaminase; BB, beta-blockers; BT, body temperature; BUN, blood urea nitrogen; Bwt, body weight; Ca, calcium; CAD, coronary artery disease; CCB, calcium-channel blockers; Chol, cholesterol; Cl, chloride; CO2, serum bicarbonate; Cr, serum creatinine; CV, contrast volume; DBP, diastolic blood pressure; DM, diabetes mellitus; eGFR, estimated glomerular filtration rate; Glu, glucose; GN, glomerulonephritis; Hb; hemoglobin; HCT, hematocrit; HCT, hematocrit; HTN, hypertension; INS, insulin; LC, liver cirrhosis; Na, sodium; otherOHA; oral hypoglycemic agents except metformin, sodium-glucose cotransporter 2 inhibitors, and dipeptidyl peptidate-4 inhibitors Phos, phosphate; PLT, platelet; PR, pulse rate; Prot, protein; SBP, systolic blood pressure; SBP, systolic blood pressure; UA, uric acid; WBC, white blood cells.


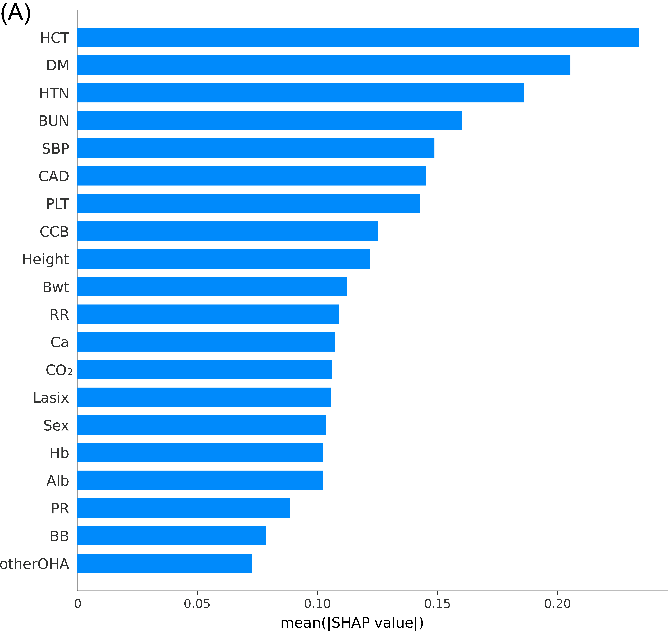

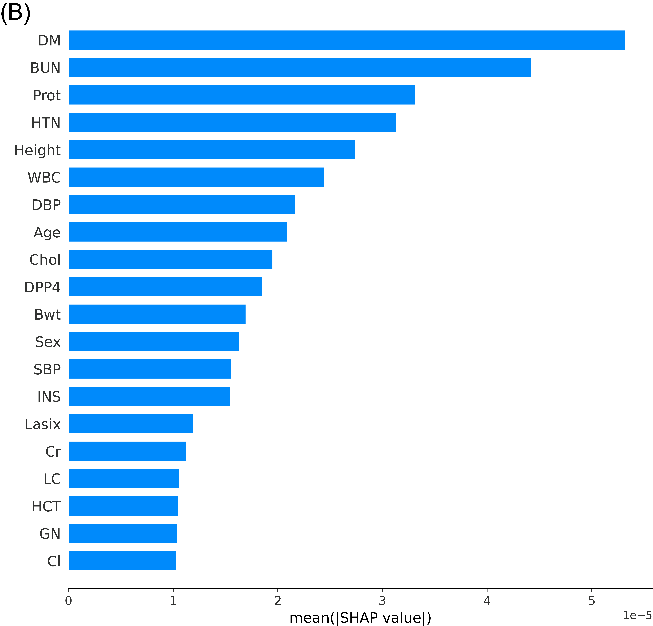


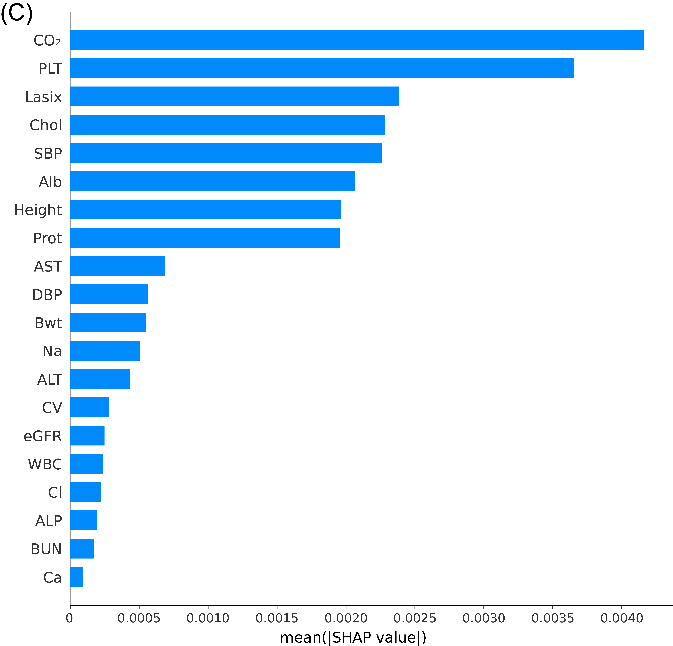

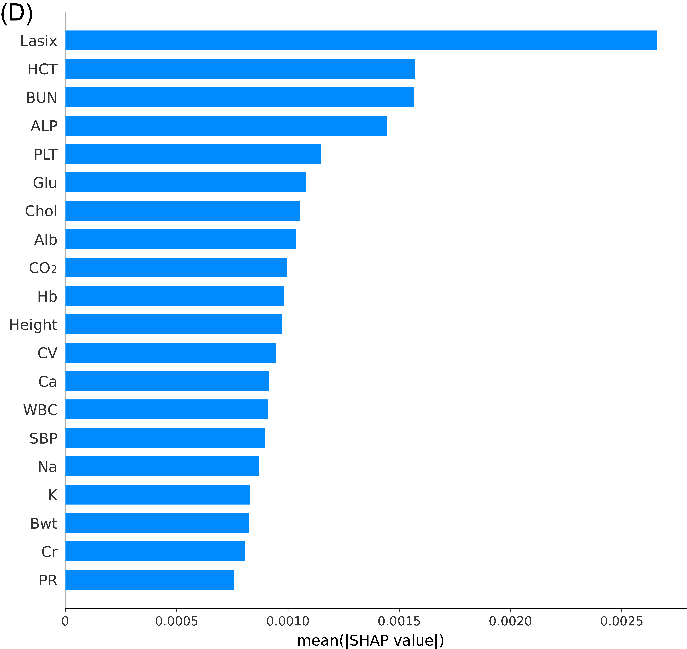


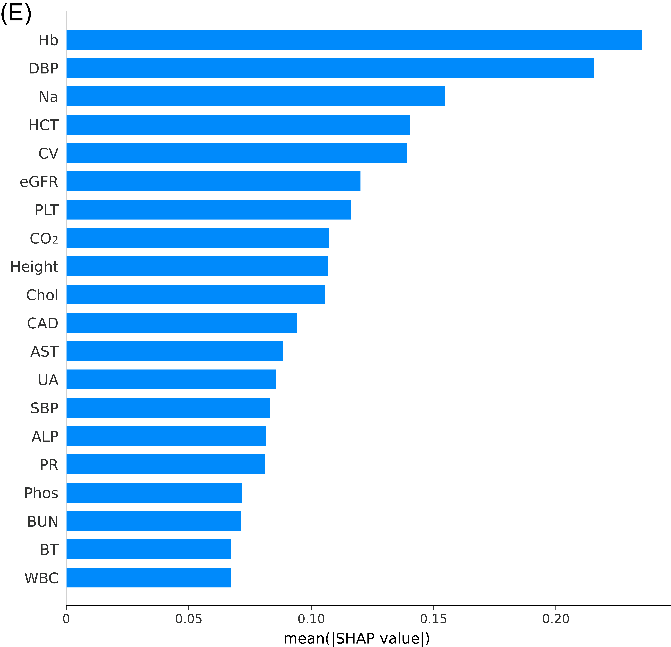

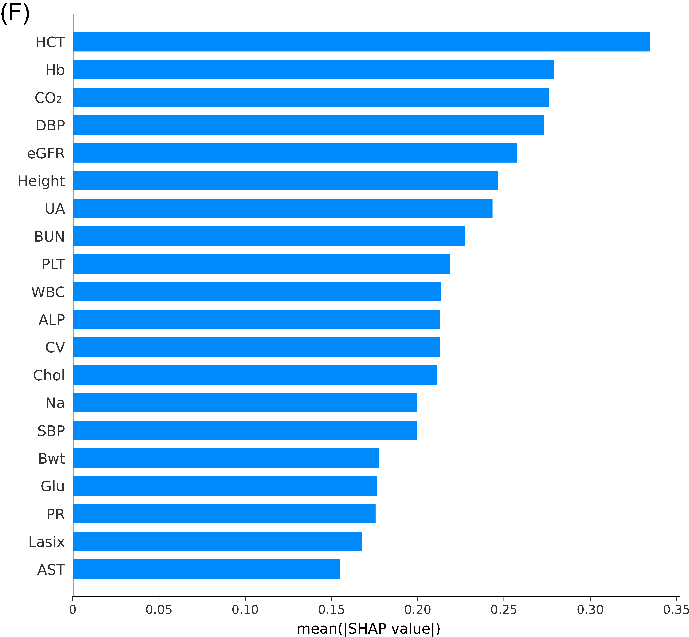

Supplement: Multimedia Appendix 12 [file medinform_v9i10e27177_app12.docx]
